# Memory-like Differentiation Enhances NK Cell Responses to Melanoma

**DOI:** 10.1158/1078-0432.CCR-21-0851

**Published:** 2021-06-29

**Authors:** Nancy D. Marin, Bradley A. Krasnick, Michelle Becker-Hapak, Leah Conant, Simon P. Goedegebuure, Melissa M. Berrien-Elliott, Keenan J. Robbins, Jennifer A. Foltz, Mark Foster, Pamela Wong, Celia C. Cubitt, Jennifer Tran, Christopher B. Wetzel, Miriam Jacobs, Alice Y. Zhou, David Russler-Germain, Lynne Marsala, Timothy Schappe, Ryan C. Fields, Todd A. Fehniger

**Affiliations:** 1 Division of Oncology, Department of Medicine, Washington University School of Medicine, Siteman Cancer Center, St. Louis, Missouri.; 2 Section of Surgical Oncology, Department of Surgery, Washington University School of Medicine, Siteman Cancer Center, St. Louis, Missouri.

## Abstract

**Purpose:**

Treatment of advanced melanoma is a clinical challenge. Natural killer (NK) cells are a promising cellular therapy for T cell–refractory cancers, but are frequently deficient or dysfunctional in patients with melanoma. Thus, new strategies are needed to enhance NK-cell antitumor responses. Cytokine-induced memory-like (ML) differentiation overcomes many barriers in the NK-cell therapeutics field, resulting in potent cytotoxicity and enhanced cytokine production against blood cancer targets. However, the preclinical activity of ML NK against solid tumors remains largely undefined.

**Experimental Design:**

Phenotypic and functional alterations of blood and advanced melanoma infiltrating NK cells were evaluated using mass cytometry. ML NK cells from healthy donors (HD) and patients with advanced melanoma were evaluated for their ability to produce IFNγ and kill melanoma targets *in vitro* and *in vivo* using a xenograft model.

**Results:**

NK cells in advanced melanoma exhibited a decreased cytotoxic potential compared with blood NK cells. ML NK cells differentiated from HD and patients with advanced melanoma displayed enhanced IFNγ production and cytotoxicity against melanoma targets. This included ML differentiation enhancing melanoma patients' NK-cell responses against autologous targets. The ML NK-cell response against melanoma was partially dependent on the NKG2D- and NKp46-activating receptors. Furthermore, in xenograft NSG mouse models, human ML NK cells demonstrated superior control of melanoma, compared with conventional NK cells.

**Conclusions:**

Blood NK cells from allogeneic HD or patients with advanced melanoma can be differentiated into ML NK cells for use as a novel immunotherapeutic treatment for advanced melanoma, which warrants testing in early-phase clinical trials.

**Translational Relevance**

Natural killer (NK)-cell therapy is a rational treatment for patients with checkpoint blockade–resistant tumors. NK cells present in the tumor microenvironment of advanced melanoma exhibit changes that include reduced activating receptors and functionality. We hypothesized that the increased expression of activating receptors, reduction in sensitivity to inhibitory killer Ig-like receptors (KIR), and augmented cytotoxic effector molecules in memory-like (ML) NK cells will augment NK-cell anti-melanoma responses. Indeed, this preclinical study demonstrates enhanced response by ML NK cells against autologous and allogeneic melanoma targets *in vitro* and *in vivo*. Mechanistically, this depended upon NKG2D and NKp46 recognition. As allogeneic ML NK cells have been safely used as a cellular therapy for leukemia, these data provide proof-of-principle to test allogeneic or autologous ML NK cellular therapy in patients with advanced melanoma.

## Introduction

Melanoma is an aggressive cancer that remains a clinical challenge due to the high risk of recurrence and rapidly progressive course seen in aggressive variants of the disease (1). Targeted and immunotherapeutic approaches have revolutionized the treatment of advanced melanoma (2). Particularly, the introduction of the immune checkpoint blocking antibodies against CTLA-4 and PD-1 have significantly improved the survival of patients with advanced or metastatic melanoma (3–5). However, despite these advances, >50% of patients are refractory to checkpoint blockade (6). Furthermore, of patients who initially respond to checkpoint blockade, approximately 50% relapse (7), highlighting the need to explore alternative immunotherapies.

Natural killer (NK) cells are cytotoxic innate lymphoid cells (ILC) that display potent effector responses against a wide variety of tumor cells (8). NK cells contribute to cancer immunoediting and are frequently deficient or dysfunctional in patients with cancer (9). A prospective clinical trial demonstrated that low NK-cell function in blood predicted an increased risk of subsequently developing cancer and reduced NK-cell numbers correlate with poor outcomes (10). NK cells mediate cytotoxic functions against targets by perforin/granzymes released from a cytotoxic synapse or via death receptor ligands. After activation, NK cells secrete cytokines (e.g., IFNγ, TNF) and chemokines (e.g., MIP1α) that modulate the function and trafficking of other immune cells, promoting an inflamed tumor microenvironment. NK-cell response to a target cell is regulated by the balance of signals received through activating receptors (that recognize stress-induced ligands) and inhibitory receptors [that recognize MHC class I (MHC-I)], with functionality tuned by cytokine receptors (11, 12). Thus, NK cells recognize tumor targets in a fundamentally different way than T cells. This feature allows NK cells to uniquely circumvent immune evasion mechanisms involving reduced MHC-I (13), as occurs in some tumors resistant to immune checkpoint inhibitors. This supports the idea that NK cell–based immunotherapy represents a promising complementary innate immunotherapy for treating patients with advanced melanoma who are resistant to T cell–based immunotherapies.

Prior studies of patients with melanoma have shown that NK cells present in tumors display an altered phenotype, which may contribute to a hypofunctional NK-cell compartment (14, 15). These tumor-infiltrating NK cells display reduced expression of activating receptors and reduced production of cytotoxic molecules which correlated with poor prognosis (16, 17). Therefore, therapeutic strategies that rescue NK-cell functionality resulting in enhanced anti-melanoma response represent an exciting approach to investigate for patients with advanced melanoma who are refractory to conventional melanoma therapeutics.

Our group and others have demonstrated that human NK cells activated briefly through IL12, IL15, and IL18 receptors differentiate into memory-like (ML) NK cells that display enhanced functionality including antitumor responses (18–22). ML NK cells have shown to be safe and induce clinical remissions in patients with relapsed/refractory acute myeloid leukemia (AML; refs. 21, 23), and these cells exhibit a variety of attributes that confers superior ability to recognize and control tumor cells (24). ML NK cells can ignore signals from some inhibitory receptors [inhibitory killer Ig-like receptors (iKIR)], and exhibit increased expression of the activating receptors NKG2D, DNAM-1, and NKp46 (21, 23), suggesting that they may be effective in recognizing additional tumor types, such as melanoma. Indeed, NKG2D and NKp46 were shown to trigger NK cytokine production and degranulation in response to melanoma targets (25) and ligands for these activating receptors are expressed by melanoma cells (26). Because these activating receptors are significantly upregulated in ML NK cells compared with control NK cells (21), this might suggest that they contribute to the control of melanoma cells by ML NK cells.

In animal models, murine IL12/15/18-induced ML NK cells were demonstrated to have enhanced response to the melanoma cell line B16 (22). However, the activity of human ML NK cells to control human melanoma has not been studied. Here, using *in vitro* and an *in vivo* preclinical studies, we tested the hypothesis that autologous and allogeneic ML NK cells possess a superior ability to recognize and control melanoma, compared with conventional control (c)NK cells.

## Materials and Methods

### Healthy donors and patient samples

Human peripheral blood mononuclear cells (PBMC) from anonymous healthy donors (HD) were obtained from leukoreduction filters after platelet apheresis and isolated by Ficoll centrifugation. For selected experiments (and as indicated on each figure legend), NK cells from HD were purified using RosetteSep (STEMCELL Technologies; routinely ≥95%CD56^+^CD3^−^). Sixteen patients with advanced (stage IIC–IV) melanoma were enrolled in a Protocol Review and Monitoring Committee and Institutional Review Board–approved protocol for tissue collection. PBMC and samples from metastatic melanoma lesions (Met) were collected from all patients (Supplementary Table S1). Uninvolved lymph nodes (ULN; defined as grossly normal-appearing lymph nodes within a regional lymph node dissection, confirmed on final pathology microscopic analysis) were also collected in 7 patients. Resected tumor specimens and ULN were mechanically and enzymatically digested to prepare single-cell suspensions as described previously (27). Frozen PBMC and single-cell suspensions from Met and ULN were thawed for mass cytometry studies. For mass cytometry studies, HD were in the same age range of patients with advanced melanoma (median, 58.8; range, 42–78 years). ML NK cells were generated from PBMC as described previously (21). Briefly, 3–5 × 10^6^ per mL of cells were preactivated with IL12 (10 ng/mL, BioLegend), IL15 (50 ng/mL, Miltenyi Biotec), and IL18 (50 ng/mL, InvivoGen) for 12 to 16 hours at 37°C/5% CO_2_. The same number of cells cultured in 1 ng/mL IL15 (control NK) served as control. Cytokines were extensively washed (three times with 50 mL PBS + 0.5% human albumin) to remove IL12, IL15, and IL18 and then differentiated *in vitro* for 6–7 days in presence of low-dose IL15 (1 ng/mL) to sustain cell survival.

### Cell lines and primary tumor characterization

K562 cells (ATCC, CCL-243) were obtained in 2008, viably cryopreserved, and thawed for use in these studies. K562 cells were maintained for <2 months at a time in continuous culture according to ATCC instructions. The human melanoma cell lines DM6 [MHC-I chain-related A and B (MICA/B) and UL-binding proteins 1/2/3 (ULBP 1/2/3)], DM6-RhoC-Luc, and M14 (MICA/B-neg) were a kind gift from Beatriz Carreno (28). All lines were *Mycoplasma* free as tested by MycoAlert Plus Mycoplasma Detection Kit (Lonza Rockland, Inc.). Patient-derived tumor cell lines were generated from single-cell suspension prepared from tumor specimens, maintained in RPMI + 10% FBS and penicillin/streptomycin, and used within 10 passages. Further characterization of patient-derived cell lines was done by staining for SOX10, Melan A, and S100 (Supplementary Data).

### NK-cell functional assays and blocking experiments

Control and ML NK cells were restimulated with K562, DM6, M14 cells, or autologous melanoma targets for 6 hours in presence of 1 ng/mL rhIL15. Effector/target (E:T) ratio was 5:1, unless otherwise indicated. When indicated, control and ML NK cells were preincubated with anti-NKG2D (2.5 μg/mL; BioLegend) and anti-NKp46 (2.5 μg/mL; BioLegend) blocking antibodies 30 minutes before coincubation with tumor targets. Cells were stained as described previously (21). Data were acquired on a Gallios flow cytometer (Beckman Coulter) and analyzed using FlowJo software (Tree Star v10.6).

### NK-cell cytotoxicity against melanoma targets

Cytotoxicity of control and ML NK cells was assessed in a standard 4-hours ^51^Cr release assays as described previously (29). Blocking or isotype control antibodies were added to serially diluted effectors to a final concentration of 2.5 μg/mL prior to the addition of labeled targets. Percent specific lysis was calculated by: (cpm experimental − cpm spontaneous release)/(cpm max release − cpm spontaneous release)*100. Specific killing of control and ML NK cells was also evaluated using IncuCyte Live-cell Analysis system. A total of 10,000 GFP-expressing DM6-RhoC-Luc cells were incubated for 2 hours in a 96-well plate and imaged prior to the addition of the NK cells at a 5:1 E:T ratio. Real-time images were captured every 3 hours and up to 72 hours and analyzed using the Incucyte software. Data are presented as green object counts (GFP^+^ DM6 melanoma cells).

### Adoptive transfer of control and ML NK cells into NSG mice

NOD-scid IL2Rγ null (NSG) mice (6–10 weeks old; Jackson Laboratories) were irradiated with 250 cGy and injected with 1 × 10^5^ DM6-RhoC-Luc (intraperitoneal) at day 0. Five million control or preactivated NK cells generated as described in Fig. 3A (21) were injected intraperitoneally in tumor-bearing mice at day 1. Mice were confirmed for the presence of tumor by bioluminescent imaging (BLI) before intraperitoneal NK-cell injection and imaged weekly for the duration of the experiment. rhIL2 (50,000 IU) was administered intraperitoneally every other day to support transferred cells. BLI was performed on an amiHT optical imaging system (1–60 seconds exposure, bin8, FOV12 cm, open filter) as described previously (30).

### Single-cell mass cytometry and analysis

PBMC from HD (*N* = 15) and advanced melanoma (*N* = 11) patients as well as metastasis-infiltrating (Met) NK cells were assessed by mass cytometry using a panel that encompassed 38 lineage, maturation, costimulatory, inhibitory, as well as function-related markers (23). Clinically uninvolved ULN were analyzed as reference for the tissue resident NK-cell phenotype. All mass cytometry data were collected on a Helios mass cytometer (Fluidigm) and analyzed using Cytobank as described previously (31). An established mass cytometry panel that included lineage and maturation markers, inhibitory and activating receptors, and function-associated molecules was utilized (Supplementary Table S2). NK cells were identified as shown in Supplementary Fig. S1A. For the comparative analysis of circulating and tissue NK cells, CD127^+^ cells were gated out to exclude ILCs and CD94 was included as a surrogate marker to identify the less mature NK-cell subsets CD56^bright^ and CD56^dim^CD16^−^ cells.

### Statistical analysis

The data are represented as Box and Whiskers plots showing interquartile range with minimum and maximum values or bars that represent mean ± SEM. Differences between groups were assessed using unpaired *t* test or ANOVA as appropriate. Linear mixed models were used for repeatedly measured data, followed by Tukey *post hoc* test for multiple comparisons. The data analyses were performed using Prism v8. *P* values are based on two-tailed tests and *P* < 0.05 was considered significant; *, *P* < 0.05; **, *P* < 0.01; ***, *P* < 0.001; ****, *P* < 0.0001.

## Results

### NK cells infiltrate metastatic melanoma and exhibit reduced cytotoxic potential

**Figure 1.**
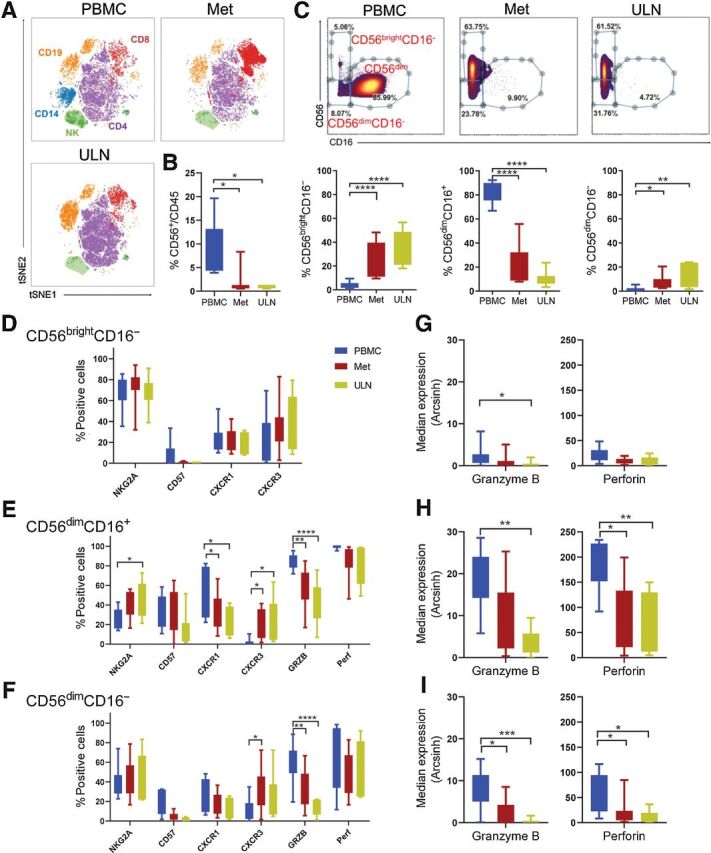
NK cells infiltrating metastatic melanoma are mostly immature CD56^bright^ and less frequently mature CD56^dim^ NK cells that exhibit reduced cytotoxic molecules. **A,** Representative example of a viSNE map showing the distribution of main immune subsets in PBMC, metastatic (Met), and ULN from a patient with advanced melanoma. **B,** Percent of NK cells (CD56^+^CD3^−^) within CD45^+^ cells. **C,** Three NK-cell subsets were identified based on CD56 and CD16 expression: CD56^bright^, CD56^dim^CD16^+^, and CD56^dim^CD16^−^ and their frequency within total NK cells is shown. **D,** NK cells were analyzed for the expression of NK cell–associated markers using mass cytometry (see Supplementary Table S2). **D–F,** Maturation markers (NKG2A, CD57), chemokine receptors (CXCR1, CXCR3) and function-related molecules (GRZB, Perf) in CD56^bright^, CD56^dim^CD16^+^ and CD56^dim^CD16^−^ assessed by mass cytometry. **G–I,** Median of GzmB and perforin expression in NK cells from the three tissue sources. Box and Whiskers plots show interquartile range with min and max values. Two-way ANOVA test, mixed effects model with Tukey *post hoc* test. *, *P* < 0.05; **, *P* < 0.01; ***, *P* < 0.001; ****, *P* < 0.0001. *n* = 11 PBMC, *n* = 11 Met, *n* = 7 ULN.

Phenotypic and functional alterations have been described in blood and tumor-infiltrating NK cells from patients with cancer, including patients with advanced melanoma (14, 15, 17). To better understand NK cells in patients with advanced melanoma, mass cytometry was used to evaluate blood and Met NK cells. Patients with stage IIIC/IV advanced melanoma were studied (*N* = 11), and their clinical characteristics described in Supplementary Table S1. NK cells, monocytes, B cells, and CD4^+^ and CD8^+^ T cells were identified in PBMC, Met and ULN but only regulatory CD4^+^ T cells were found to be significantly higher in Met compared with PBMC (Fig. 1B; Supplementary Fig. S1B–S1D). NK cells were found within the Met and ULN and were predominantly CD56^bright^ NK cells, as previously reported for secondary lymphoid tissues (32). CD56^dim^CD16^+^ NK cells were more frequent in blood (Fig. 1C) although a small percentage was also detected in both Met and ULN. NK cells in tissue expressed high levels of CD69 and decreased levels of CD62L, consistent with a tissue (lymph node) resident phenotype (Supplementary Fig. S2). Frequencies of both total NK cells and NK-cell subsets were not different between Met and ULN (Fig. 1C). Analysis of maturation, chemokine receptors, and function-related markers showed no differences in the phenotype of CD56^bright^ NK cells from the three tissue sources (Fig. 1D and G). In contrast, CD56^dim^CD16^+^ NK cells infiltrating tissue exhibited diminished cytotoxic potential indicated by a reduced expression of granzyme B (GzmB) and perforin (Fig. 1E and H). In addition to the conventional CD56^bright^ and CD56^dim^CD16^+^ NK-cell subsets, we also detected the aberrant subset of CD56^dim^CD16^−^ NK cells that have been previously reported in patients with chronic viral infection and cancer (33, 34). CD56^dim^CD16^−^ NK cells infiltrating tumor tissue also exhibited reduced expression of GzmB and perforin compared with paired PBMC (Fig. 1F and I). CD56^dim^CD16^+^ NK cells from ULN expressed higher levels of NKG2A suggesting a less differentiated phenotype compared with their counterpart in blood. The high CXCR1 and low CXCR3 expression that mark mature CD56^dim^CD16^+^ NK cells in blood (35, 36) also contrasted with the phenotype of tissue-resident NK cells, which expressed low CXCR1 and high CXCR3 (Fig. 1F). These data are consistent with previous findings showing that immature CD56^bright^CD16^−^ NK cells are a predominant population in advanced melanoma lymph nodes (34). Thus, NK cells infiltrate advanced melanoma environments and exhibit a reduced cytotoxic potential with decreased expression of GzmB and perforin, a defect that could be addressed by adoptive NK-cell therapies.

### Circulating NK cells from patients with advanced melanoma exhibit an altered repertoire of activating receptors and decreased expression of granzyme and perforin

Next, a comparative phenotypic analysis of blood NK cells from 11 patients with advanced melanoma and 15 HD was performed to define the NK-cell multidimensional phenotype in patients with advanced melanoma. No differences in the frequency of total NK cells were observed between advanced melanoma and HD. However, in patients with melanoma there were reduced CD56^dim^CD16^−^ and increased CD56^dim^CD16^+^ NK-cell frequencies (Supplementary Fig. S3A and S3B). A multidimensional analysis showed that CD56^bright^ NK cells from patients with advanced melanoma exhibited decreased expression of the activating receptors NKp44 and DNAM-1, with no other major changes (Supplementary Fig. S3C and S3F). Similarly, CD56^dim^CD16^+^ NK cells also exhibited reduced DNAM1 as well as GzmB and perforin expression (Supplementary Fig. S3D and S3G) while modest changes in the activating receptors NKG2D and NKp46 expression were found in CD56^dim^CD16^−^ NK cells from advanced melanoma compared with HD (Supplementary Fig. S3E and S3H). These data, in concert with prior studies, suggest that blood NK cells from patients with advanced melanoma have reduced expression of activating receptors and cytotoxic potential. This altered functionality was more pronounced in advanced melanoma–resident NK cells suggesting that approaches to enhance anti-melanoma activity of NK cells will be required for an effective autologous response.

### ML NK cells from HD PBMC exhibit enhanced ability to control melanoma compared with conventional NK cells

**Figure 2.**
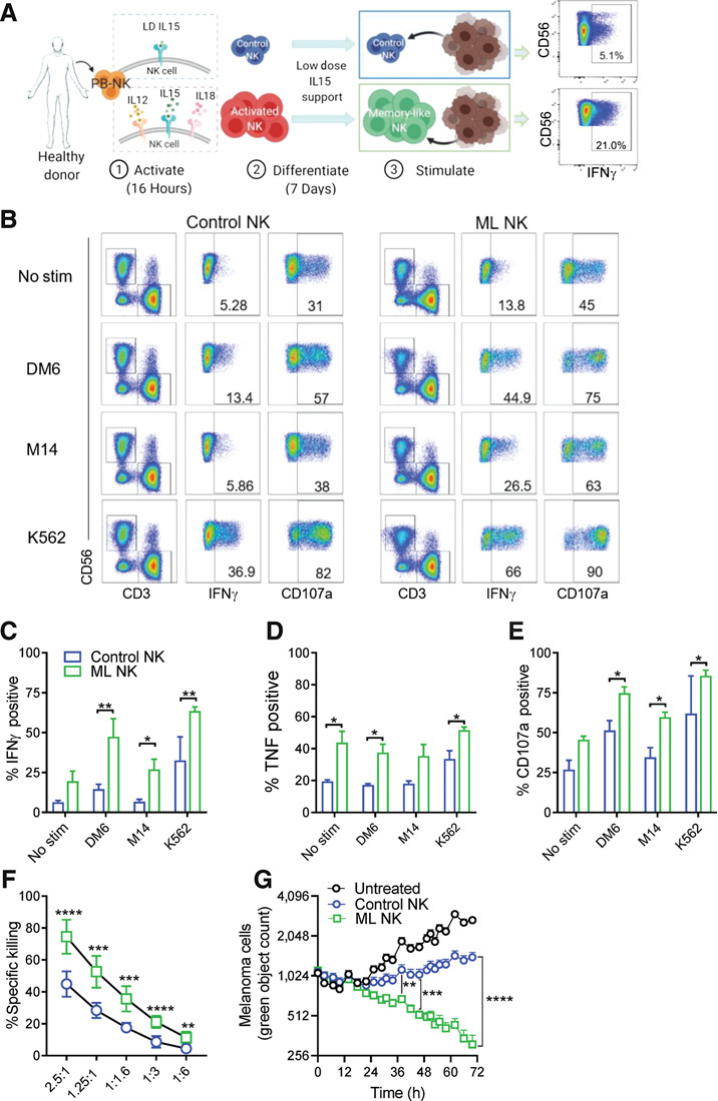
ML NK cells from normal donors exhibit enhanced ability to control melanoma compared with conventional NK cells. **A,** PBMC-derived NK cells from HD were activated with IL12/IL15/IL18 (or IL15 alone as control) for 16–18 hours (1). Activated NK cells were differentiated into ML NK cells in vitro during 7 days in presence of low-dose IL15 (2). Upon a second stimulation (3), ML NK cells exhibited enhanced responses against tumor target and cytokines, compared with control or conventional NK cells. **B,** Representative flow cytometry and gating strategy to evaluate cytokine secretion and degranulation (CD107a^+^ cells) of control and ML NK cells stimulated with melanoma cell lines (DM6, M14) and K562 cells. Numbers indicate percentage of positive cells. Summary data of IFNγ (**C**), TNF (**D**), and degranulation of control (blue) and ML (green) NK cells (**E**) restimulated 6 hours *in vitro* with DM6, M14, and K562 cells. Cells lines were used at a 10:1 E:T ratio. ML NK cell exhibited superior killing ability against DM6 compared with control NK cells in a standard 4-hours ^51^Cr release assay (**F**) and a 72-hour Incucyte assay (**G**). Purified NK cells were used for the cytotoxic assays. Bars represent mean ± SEM. Two-way ANOVA test. *, *P* < 0.05; **, *P* < 0.01; ***, *P* < 0.001; ****, *P* < 0.0001. *n* = 3 in **C–E**, *n* = 9 in **F**. **G** shows a representative experiment out of three independent experiments.

ML NK cells exhibit potent cytotoxic functions and enhanced ability to produce cytokines upon *in vitro* or *in vivo* restimulation with tumor targets (21, 22, 37). However, activity of human ML NK responses against solid tumors remains largely undefined. To address this, we evaluated the ability of ML NK cells from HD to respond and kill melanoma cells. ML NK cells from HD were generated following IL12, IL15, and IL18 receptor activation using PBMC as described previously (Fig. 2A), allowed to differentiate, and then restimulated with the human melanoma cell lines DM6 and M14. These were compared with conventional (cNK, control) NK cells maintained in low-dose IL15 for survival. Despite the high expression of human NKG2D ligands MICA/B and ULBP1/2/3 (29) in DM6 cells, only a modest increase in IFNγ production and CD107a expression (as a surrogate for degranulation) was evident in control NK cells (Fig. 2B–E). No significant induction of IFNγ was observed in control NK cells upon stimulation with M14 cells, which have reduced MICA/B expression. In contrast, ML NK cells from the same HD had significantly increased IFNγ, TNF, and CD107a expression after restimulation with DM6 and M14 melanoma cells (Fig. 2B–E). Superior cytokine response and degranulation of ML NK cells was also significant when purified NK cells were used instead of PBMC (Supplementary Fig. S4A–S4C). Consistent with increased degranulation, ML NK cells from HD also exhibited significantly increased and sustained cytotoxicity, thereby eliminating melanoma target cells even at low E:T ratios (Fig. 2E and F). These results support a significantly enhanced ability of ML NK cells to control melanoma targets *in vitro*.

### ML NK cells differentiated from patients with advanced melanoma exhibit enhanced ability to control autologous tumor

**Figure 3.**
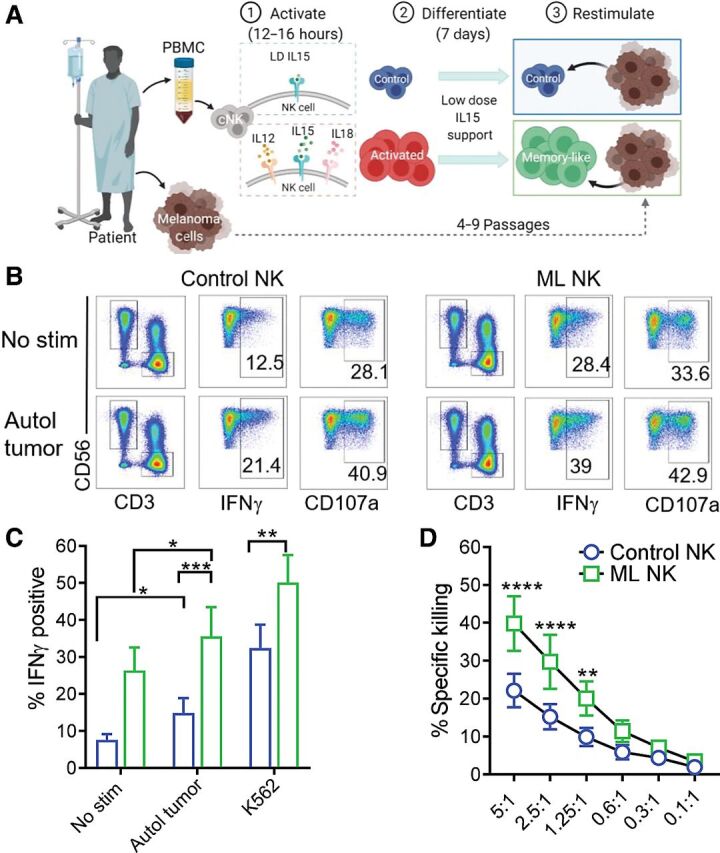
Memory-like NK cells from patients with advanced melanoma exhibit enhanced ability to control autologous tumor. **A,** PBMC containing patient NK cells were preactivated as indicated in Fig. 2A. Melanoma cell lines generated from metastatic tissue were used as target cells for the functional assays. **B,** Representative flow cytometry plots of control and ML NK cells from a patient with advanced melanoma stimulated with autologous tumor. Numbers indicate percentage of positive cells. ML NK cells (green) from patients with melanoma patients display superior ability to produce IFNγ upon stimulation with autologous tumor (5:1 E:T ratio) compared with control NK cells (blue; **C**). **D,** ML NK cells from patients with advanced melanoma exhibit superior ability to kill autologous melanoma targets compared with control NK cells at different E:T ratios. Killing was evaluated in a standard 4-hours ^51^Cr release assay. Bars represent mean ± SEM from all patients. Two-way ANOVA with Tukey *post hoc* analysis. *, *P* < 0.05; **, *P* < 0.01; ***, *P* < 0.001; ****, *P* < 0.0001. *n* = 7.

We next asked whether ML NK cells could be generated from patients with advanced melanoma and whether they are able to control autologous melanoma targets. PBMC from patients with advanced melanoma (*n* = 7; Supplementary Table S1) were stimulated as described in Fig. 3A and their NK cells differentiated into ML NK cells (Fig. 3A). Autologous primary melanoma cells generated from metastatic lesions were used as targets in functional assays. For all patients, IHC staining of S100, MelanA, and SOX10 confirmed these cell lines to be metastatic melanoma (Supplementary Fig. S5). When restimulated with autologous melanoma targets, ML NK cells exhibited a significantly increased IFNγ production compared with control NK cells (Fig. 3B and C). In contrast to ML NK cells from HD (Fig. 2), autologous ML NK cells from patients with advanced melanoma also exhibited increased baseline IFNγ production. ML NK cells from patients with advanced melanoma also exhibited a significant increase in specific killing of autologous melanoma cells compared with control NK cells, even at low E:T ratios (Fig. 3D). As expected, there were no significant differences in CD107a in ML NK cells versus control NK cells when cells were restimulated with autologous melanoma targets or K562 cells (Supplementary Fig. S6A and S6B). These data suggest that the ML NK cells can be generated from patients with advanced melanoma and that the ML differentiation program restores IFNγ production and cytotoxicity against autologous advanced melanoma targets.

### Enhanced response of ML NK cells from patients with advanced melanoma against advanced melanoma targets are NKG2D and NKp46 dependent

**Figure 4.**
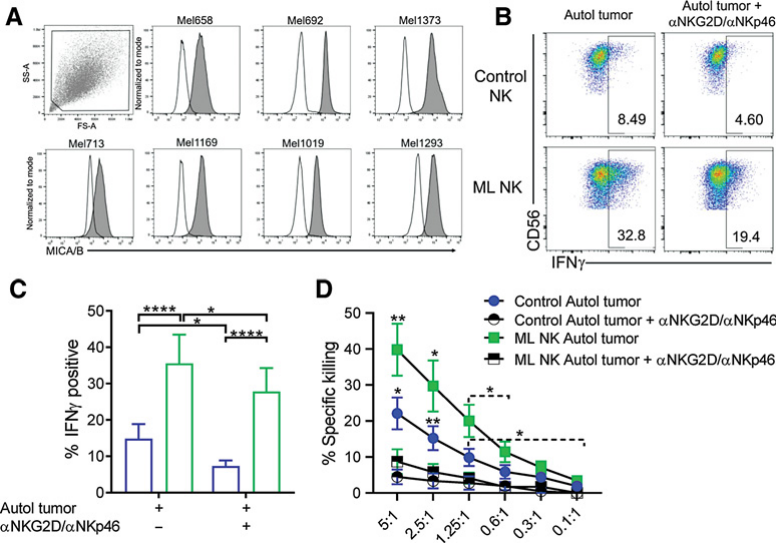
Enhanced ML responses are partially dependent on NKG2D and NKp46. **A,** Expression of the NKG2D ligand MICA/B in primary tumors. Patient-derived control and ML NK cells were stimulated for 6 hours with autologous tumor with or without αNKG2D (2.5 μg/mL) plus αNKp46 (2.5 μg/mL) blocking antibodies and the frequency of IFNγ was evaluated by flow cytometry. Representative flow cytometry dot plots (**B**) and summary data (**C**) showing reduction in IFNγ production by control and ML NK cells after NKG2D and NKp46 blockade. **D,** Patient-derived control and ML NK cells were stimulated in the presence of autologous tumor with or without blocking antibodies and specific killing was measured by ^51^Cr release assay. Bars represent mean ± SEM from all patients. Two-way ANOVA and paired *t* test *, *P* < 0.05; **, *P* < 0.01; ***, *P* < 0.001; ****, *P* < 0.0001. *n* = 7.

NKG2D and NKp46 mediate NK responses against melanoma targets (25). Because these activating receptors are significantly upregulated in ML NK cells compared with control NK cells (21), we next evaluate their contribution to patient-derived ML NK-cell recognition of melanoma targets. For this, NKG2D and NKp46 were blocked and changes in NK-cell responses against autologous tumor were assessed. All primary melanoma cells consistently expressed the stress-induced NKG2D ligand MICA/B (Fig. 4A). IFNγ production from both patient-derived control NK and ML NK cells were significantly diminished in presence of both NKG2D and NKp46 blockade (Fig. 4B and C). These differences were not associated with reduced cell viability of control and ML NK cells (Supplementary Fig. S6C). Furthermore, degranulation but not TNF production was markedly reduced in both control NK cells and ML NK cells (Supplementary Fig. S6D–S6E). Consistent with this, the killing of autologous melanoma targets by control NK cells and ML NK cells was also significantly impaired following blockade of these activating receptors (Fig. 4D). Similar reduction in cytokine response, degranulation, and killing was found after stimulation of HD-purified NK cells with DM6, suggesting a specific effect on NK cells (Supplementary Fig. S6F–S6I). Importantly, reduced NK-cell functionality was significantly affected by simultaneous blockade of NKG2D and NKp46 rather than single blockade (Supplementary Fig. S6J and S6K). Together, these results demonstrate the critical role of NKG2D and NKp46 in the enhanced response of ML NK cells against autologous melanoma targets.

### ML NK cells control melanoma targets in an NSG xenograft model

**Figure 5.**
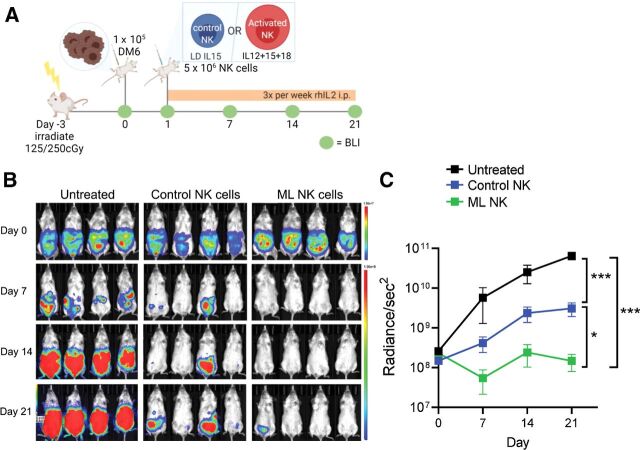
Human ML NK cells effectively control melanoma targets in a xenograft model in NSG mice. **A,** NSG mice were injected with 1 × 10^5^ (i.p.) DM6 melanoma cells expressing RhoC/*Luc* 1 day before NK-cell injection. At day 1, tumor-bearing mice were injected with 5 × 10^6^ control or ML NK cells (intraperitoneal) and the tumor burden was assessed using BLI every week (**B**). BLI images in **B** are representative from one of three independent experiments. **C,** Summary data indicate that allogenic ML NK cells control melanoma targets *in vivo* better than control NK cells. Three to seven mice per group from three independent experiments. **C,** Data from all mice from the three experiments (untreated = 11 mice, control NK cells = 16 mice, and ML NK cells = 15 mice). Two-way ANOVA - mixed-effect model with Tukey post-test. *, *P* < 0.05; ***, *P* < 0.001.

Next, human ML NK cells were evaluated for their ability to control melanoma targets *in vivo* using a human xenograft model into immunodeficient NSG mice. NSG mice were injected with 1 × 10^5^ DM6-RhoC-Luc cells (intraperitoneal), and 1 day later mice received a single injection of 5 × 10^6^ control NK cells or ML NK cells (intraperitoneal). Because NSG mice lack a homeostatic IL2/15R ligand required to support NK-cell survival and expansion, IL2 (50,000 IU/mouse) was injected every other day (intraperitoneally) to support transferred human NK cells (21) and the melanoma burden was monitored by BLI weekly (Fig. 5A). Mice receiving control NK cells were able to significantly control melanoma tumor burden compared with untreated mice, although the tumor advanced over time. In contrast, mice adoptively transferred with a single dose of ML NK cells (from the same donors) exhibited a significantly superior ability to control melanoma cells *in vivo*, with the tumor burden controlled over the duration of the experiment (Fig. 5B and C). These data indicate that a single injection of ML NK cells provided enhanced control or melanoma targets *in vivo*, compared with control NK cells.

## Discussion

Here, we demonstrated that IL12, IL15, and IL18 receptor induced ML NK cells from HD and patients with advanced melanoma exhibit an enhanced ability to attack melanoma targets. ML differentiation of patients' blood NK cells resulted in enhanced ability to produce IFNγ and kill autologous melanoma targets. We also identified NKG2D and NKp46 as a recognition mechanism underlying the enhanced functionality of ML NK cells against melanoma. Using mass cytometry and multidimensional analysis, we found that patient NK cells infiltrating tumors exhibited a dysfunctional phenotype with reduced expression of cytotoxic molecules and altered expression of chemokine receptors compared with patient-matched blood NK cells. Furthermore, circulating NK cells from patients with advanced melanoma exhibited reduced expression of activating receptors, GzmB, and perforin when compared with HD blood NK cells. Thus, the ML differentiation of NK cells from dysfunctional patients with advanced melanoma or HD represents new approaches to enhance NK-cell anti-melanoma attack and warrants further investigation as a cellular therapy.

Immune checkpoint blockade (ICB) therapy targeting PD-1 and CTLA-4 as single or combined therapy has revolutionized the treatment of patients with several cancer types including melanoma (38, 39). Blocking these inhibitory receptors enhances T cell–mediated antitumor immune responses, leading to improved clinical responses and survival of patients with advanced melanoma (38, 39). However, despite their successes, immune- and drug-related adverse effects are common and affect >50% of the patients receiving the combination therapy (38). In addition, >50% of the patients fail to ever respond and approximately 50% of those who initially respond, become resistant and eventually develop progressive disease (5, 7, 40). In addition, for those with BRAF-mutant melanoma tumors, targeted therapy alone has led to survival improvements (41); however, this is not curative. Tumor intrinsic and extrinsic mechanisms are associated with resistance to ICB including downregulation of MHC-I antigen presentation pathways by tumor cells to evade a cytotoxic T-cell antigen-specific immune response (40, 42). NK cells have emerged as a promising therapy for treating patients with cancer as they are safe and exert potent antitumor responses (24, 43). Their ability to recognize tumor cells in an antigen-independent manner allows NK cells to circumvent immune evasion mechanisms involving reduced MHC-I expression (40, 42). Thus, NK cells become a suitable therapy for treating tumors that have failed ICB, targeted, and T cell–based therapies.

The recently discovered memory properties of innate immune cells, also referred to as trained immunity, have changed the paradigm of enhanced recall responses as an exclusive feature of adaptive T and B cells. NK cells that remember their prior experiences have been described in response to haptens, viral infections, and cytokine stimulation (18, 19, 44). We and others have shown that a short-term stimulation through IL12, IL15, and IL18 receptors induce NK cells with an enhanced ability to produce IFNγ, TNF, and MIP1α in response to cytokines, activating receptors triggering or restimulation with tumor targets (19, 21, 22). Cytokine-induced ML NK-cell therapy has been tested in patients with leukemia and was both safe and effective, inducing complete remission in 47% of patients with relapsed/refractory AML (23). The first evidence demonstrating the ability of ML NK cells to reject solid tumors was reported in syngeneic mouse models by the Cerwenka laboratory (22, 37). In those studies, IL12/15/18-activated NK cells exhibited a stable and persistent enhanced ability to control B16 melanoma cells *in vivo*, in a mechanism dependent on IL2 produced by CD4^+^ T cells. More recently, Uppendahl and colleagues (45) demonstrated enhanced functionality and cytotoxicity of peritoneal NK cells against ovarian cancer cells after activation with IL12, IL15, and IL18. Our data advance these findings and show that both allogenic and autologous blood ML NK cells can effectively control human melanoma cells. The ML differentiation program also imprints NK cells with specific phenotypic and molecular changes that includes upregulation of activating receptors (NKG2D, NKp46, DNAM1), the ability to rescue unlicensed NK cells and respond to both KIR ligand matched and mismatched NK cells (20, 21), and to ignore inhibitory KIR signals (21). These attributes provide the rationale for the use of autologous or allogeneic ML NK cells as cellular therapy in melanoma. Infiltration of NK cells in melanoma correlates with improved prognosis and survival (10, 17), and multiple phenotypic and functional alterations have been described in blood and melanoma-infiltrating NK cells (14, 15, 17). Consistent with prior reports, we observed reduced NKp44 and DNAM1 expression in blood NK cells from patients with advanced melanoma (14, 25), partially accounting for the reduced anti-melanoma response. In addition, the expression of ligands for these activating receptors in melanoma cells also suggests a key role for these ligand-receptor interactions in the tumor recognition and induction of NK antitumor properties against melanoma targets (46). NKG2D and NKp46 are also expressed at low levels by NK cells from patients with melanoma (25) and they are required for an efficient tumor recognition and induction of antitumor responses against melanoma targets (46). Consistent with this, we demonstrated that the enhanced functionality of ML NK cells against melanoma is partially dependent on the recognition of melanoma cells by these activating receptors. Even though we did not test the expression of NKp46 ligands on patient-derived tumor cells, our results using DM6 cells suggest that blockade NKG2D is relatively more important than NKp46 to inhibit anti-melanoma responses of NK cells. In addition, the reduced response of ML NK cells against M14 cells which have reduced expression of NKG2D ligands (MICA/B, ULBP1/3; ref. 29) support the concept, but also indicate additional activating receptor and ligand interactions may contribute. The enhanced expression of these activating NK receptors on ML NK cells supports the premise that ML NK-cell program can rescue the hypofunctional phenotype of poor-responding NK cells from patients with advanced melanoma.

Using mass cytometry, we also identified alterations in NK cells within the tumor microenvironment. Besides CD56^bright^ and mature CD56^dim^ NK-cell subsets commonly identified in blood, CD56^dim^ NK cells also infiltrated metastatic lesions (34, 47), suggesting recirculation of mature NK cells from the periphery as part of their normal immunosurveillance. We also identified the atypical CD56^dim^CD16^−^ NK-cell population infiltrating melanomas. This subset was described to contain maturing and target cell–activated NK cells (48), and was identified as an NK-cell subset in melanoma tissue (34, 48). The relationship between CD56^dim^CD16^−^ NK cells to the better characterized CD56^bright^CD16^−^ and CD56^dim^CD16^+^ remains unclear, but because this population is increased in the setting of disease or chronic activation, may represent chronically activated/exhausted NK cells. Future studies should focus on the cellular and molecular differences of this cell type across different disease states, to HD. When comparing the three tissue sources, the significant differences associated with the location in tissue (whether Met or ULN), compared with PBMC, likely accounted for by the distinct NK cells that are present in secondary lymphoid tissue, with CD56^bright^CD16^−^ NK cells being predominant in the lymph nodes (32). There were differences based on tissue location of chemokine receptors in CD56^dim^CD16^+^ from PBMC having higher CXCR1 and lower CXCR3, compared with the lymph node samples. This was not evident in the CD56^bright^CD16^−^ subset, which may indicate distinct homing receptor use or development within the tissue.

Tumor-infiltrating NK cells have been reported to have reduced expression of cytotoxic molecules (49). Our data support those findings and demonstrated the most severe defect in the cytotoxic subsets CD56^dim^CD16^+^ and CD56^dim^CD16^−^ NK cells. Furthermore, considering the immunosuppressive nature of the tumor microenvironment, we expected to see greater differences between Met and ULN, with reduced GzmB and perforin in the Met situation. In contrast, this also appeared to be associated based on tissue location, with reduced GzmB and perforin in both Met and ULN compared with PBMC, as reported previously (17). However, both Met and ULN are within the patient with advanced melanoma and in the same lymphatic drainage, and thus may represent a general feature of NK alteration in patients with melanoma. This hypofunctional phenotype correlated with the poor responses of conventional NK cells against melanoma targets observed in this study.

Preclinical data presented in this study and by others, and data from our current clinical trials, have demonstrated superior functionality and persistence of ML NK cells *in vivo*, partially dependent on the homeostatic cytokines IL2 or IL15 (22, 23, 37). Because of the lack of a homeostatic IL2/15R ligand in NSG mice, rhIL2 was injected to support transferred human NK cells in NSG mice as initially demonstrated by the Cerwenka Laboratory (22). In addition, differences in the requirement for radiotherapy in this and other studies are likely model dependent. Utilizing a syngeneic mouse model and melanoma cell line in an immunocompetent setting, Ni and colleagues initially reported that radiotherapy was an important, potentially by providing a lymphodeplete niche for expanding ML NK cells (22). Studies by the Cooper laboratory (50) have also explored this *in vivo* in murine ML NK-cell responses in the setting of homeostatic proliferation, and radiotherapy was not required in this lymphodeplete setting. We have performed adoptive transfer of human NK cells into irradiated and nonirradiated NSG mice without major differences in their ability to persist. Furthermore, our clinical studies in leukemia did not include radiotherapy, with robust ML NK-cell responses evident *in vivo* in patients. In this setting, the flu/cy lymphodepleting chemotherapy likely takes the place of radiotherapy used in the initial murine syngeneic ML NK-cell responses to B16 melanoma and RMAS lymphoma. Finally, while the evaluation of primary melanoma patient ML NK cells *in vivo* in NSG mice would be interesting, this is technically very challenging requiring large volume blood collection or leukapheresis, which was not performed during this preclinical study.

In summary, ML NK cells exhibit an enhanced ability to control melanoma targets *in vivo* and *in vitro* in preclinical experiments. Importantly, we demonstrated that the ML differentiation programming rescues dysfunctional NK cells in patients with advanced melanoma and that patient-derived ML NK cells efficiently control autologous tumor. This provides a rationale for the use of ML NK cells as an alternative immunotherapy to treat patients with advanced melanoma resistant to standard therapies, supporting advancement of first-in-human clinical trials in melanoma.

## Authors' Disclosures

M.M. Berrien-Elliott reports personal fees and other support from Wugen during the conduct of the study; in addition, M.M. Berrien-Elliott has a patent for 15/983,275 pending and licensed to Wugen, a patent for PCT/US2019/060005 pending and licensed to Wugen, and a patent for 62/963,971 pending and licensed to Wugen. K.J. Robbins reports grants from NCI during the conduct of the study. J.A. Foltz reports grants from American Association of Immunologists and NIH T32HL007088 during the conduct of the study. In addition, J.A. Foltz has a patent for USPTO 16/966,367 and WO 2019/152387 A1 pending, licensed, and with royalties paid from Kiadis; a patent for US 63/018,108 pending, licensed, and with royalties paid from Kiadis; and canine antibody licensed to EMD Millipore. A.Y. Zhou reports other support from Gilead outside the submitted work. T.A. Fehniger reports grants from NIH during the conduct of the study. T.A. Fehniger also reports grants, personal fees, and other support from Wugen; grants from ImmunityBio, Affimed, Compass Therapeutics, and HCW Biologics; and other support from Kiadis, OrcaBio, and Indapta outside the submitted work. In addition, T.A. Fehniger has a patent for 15/983,275 pending and licensed to Wugen, a patent for PCT/US2019/06005 pending and licensed to Wugen, and a patent for 62/963,971 pending and licensed to Wugen. No disclosures were reported by the other authors.

## Supplementary Material

Supplementary Data Supplementary methods and tables

Supplementary Fig S1 Mass cytometry analysis and main immune cell subsets in PBMC, Met and ULN from AM patient samples.

Supplementary Fig S2 Infiltrating NK cells exhibit a tissue resident phenotype.

Supplementary Fig S3 Phenotypic analysis of blood NK cells from AM patients and healthy donors.

Supplementary Fig S4 Cytokine production and degranulation of purified NK cells from normal donors

Supplementary Fig S5 Immunohistochemistry staining of S100, Melan A and SOX10 in patient derived cell lines

Supplementary Fig S6 Cytokine production and degranulation of control and ML NK cells from AM patients and normal donors and effect of antibody blockade.
