# Supplementary material for: Memory-like Differentiation Enhances NK Cell Responses to Melanoma
Source: Clin Cancer Res. 2021 Jun 29;27(17):4859–69. doi: 10.1158/1078-0432.CCR-21-0851 (PMC8416927; doi:10.1158/1078-0432.CCR-21-0851)
Supplement: Supplementary Data — Supplementary methods and tables [file 10780432ccr210851-sup-261875_2_supp_7154413_qtwgkp.docx]

**Supplementary Materials**

**Supplementary Materials and Methods:**

*Patient derived cell lines*

**Supplementary Results:**

Supplementary Fig. S1. Mass cytometry analysis and main immune cell subsets in PBMC, Met and ULN from AM patient samples.

Supplementary Fig. S2. Infiltrating NK cells exhibit a tissue resident phenotype.

Supplementary Fig. S3. Phenotypic analysis of blood NK cells from AM patients and healthy donors

Supplementary Fig. S4. Cytokine production and degranulation of purified NK cells from normal donors.

Supplementary Fig. S5. Immunohistochemistry staining of S100, Melan A and SOX10 in patient derived cell lines.

Supplementary Fig. S6. Cytokine production and degranulation of control and ML NK cells from AM patients and normal donors and effect of antibody blockade.

Supplementary Table S1. Demographic and clinical data of advanced melanoma patients

Supplementary Table S2. List of antibodies used for mass cytometry characterization of conventional NK cells.

**Supplementary Materials and Methods**

*Patient derived cell lines*

Patient-derived tumor cell lines were generated from a single cell suspension prepared from tumor specimens. Briefly, tumor tissue was mechanically processed into small pieces followed by enzymatic digestion. Enzymatic dissociation media (EDM) was prepared as follows: 1g collagenase (Sigma C5138-1G), 0.1g DNAse I (Type IV; Sigma D5025), 10 mL HEPES (10mM), 2500U/1L Hyaluronidase (Sigma H6254) in 1L RPMI. The suspension was filtered over a 100 micron filter (Sigma), and the flow-through was plated in a single cell layer. Approximately 1 million cells from the filtered single cell digest was plated onto sterile 25cm2 tissue culture flasks (Sigma). Non-adherent cells were removed 24 hours after initial plating. Every 2-3 days non adherent cells were removed, and the cells were then passaged in 1-3 weeks using 0.25% Trypsin-EDTA (Gibco) once ~75% confluent. Cell lines were passaged at least 3 times prior to use and no more than 10 times. Cell lines were maintained in RPMI + 10% FBS and penicillin/streptomycin.

Further characterization of patient derived cell lines was done by staining for SOX10, Melan A and S100. Briefly, 33,000 cells in 75ul RPMI media were grown overnight on a microscopy slide (Superfrost ®PLUS, ThermoScientific) and washed with PBS (Hyclone, GE Healthcare) before fixation in 95% ethanol at 4°C. Then, slides were stained with Hematoxylin & Eosin, along with SOX-10 (Rabbit polycolonal Ab, Cell Marque-Millipore Sigma), Mart 1 (Clone M2-7C10, Cell Marque-Millipore Sigma), and S100 (4C4.9, Roche, Switzerland). Staining was performed according to manufacture instructions. The Zeiss AxioObserver D1 inverted microscope was used (Carl Zeiss Inc. Thrownwood, NY) equipped with an Axiocam 503 color camera. Images were acquired with 20X EC Plan-Neofluar (NA 0.5) objective using the ZEN 2 (blue edition) software. Staining was reviewed by author BAK while both melanoma identity and cellularity was reviewed by a board certified dermatopathologist.

**Supplementary Table S1.** Demographic and clinical data of advanced melanoma patients

**Supplementary Table S2.** List of antibodies used for mass cytometry characterization of conventional NK cells.

**
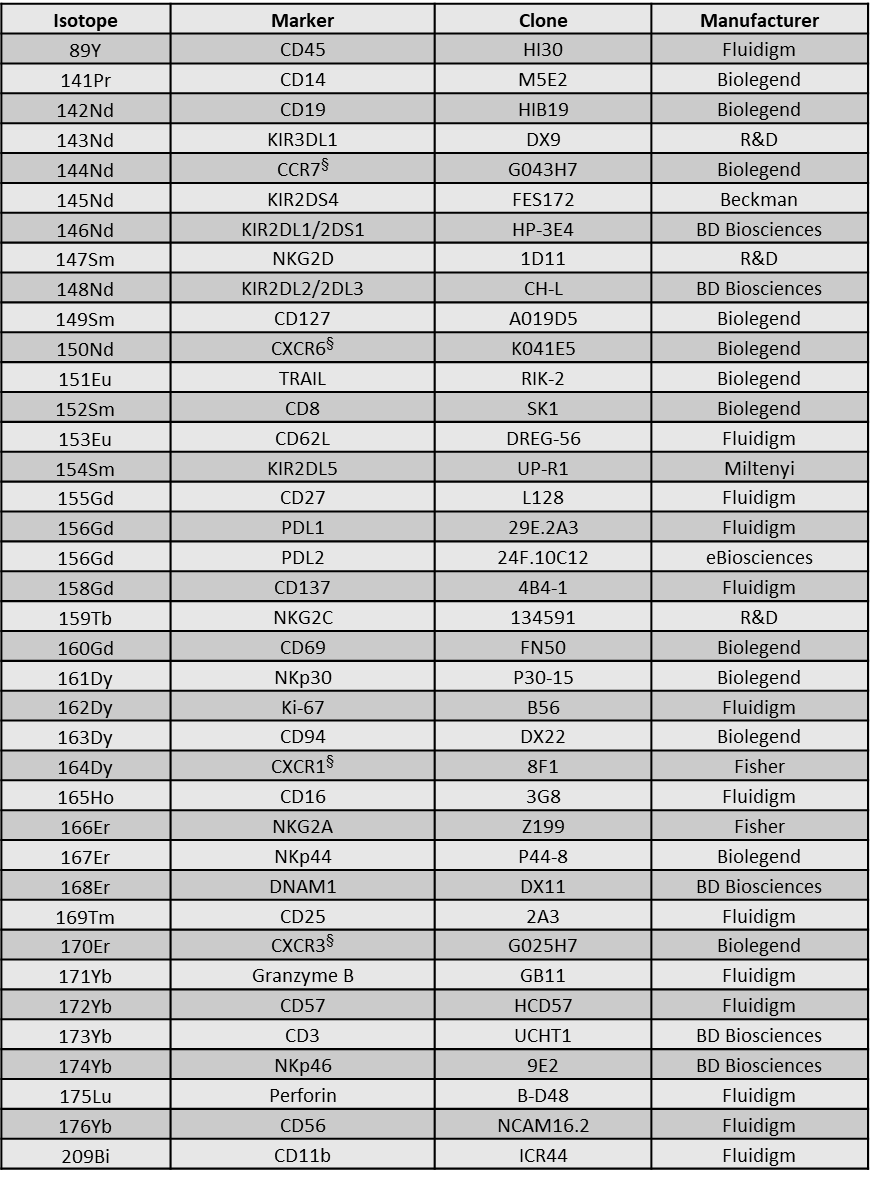
**

^§^Markers excluded in the analysis to compare circulating NK cells from HD and AM patients
