# Supplementary material for: Memory-like Differentiation Enhances NK Cell Responses to Melanoma
Source: Clin Cancer Res. 2021 Jun 29;27(17):4859–69. doi: 10.1158/1078-0432.CCR-21-0851 (PMC8416927; doi:10.1158/1078-0432.CCR-21-0851)
Supplement: Supplementary Fig S1 — Mass cytometry analysis and main immune cell subsets in PBMC, Met and ULN from AM patient samples. [file 10780432ccr210851-sup-261875_2_supp_7159133_qx1f92.pdf]

Supplementary Figure 1

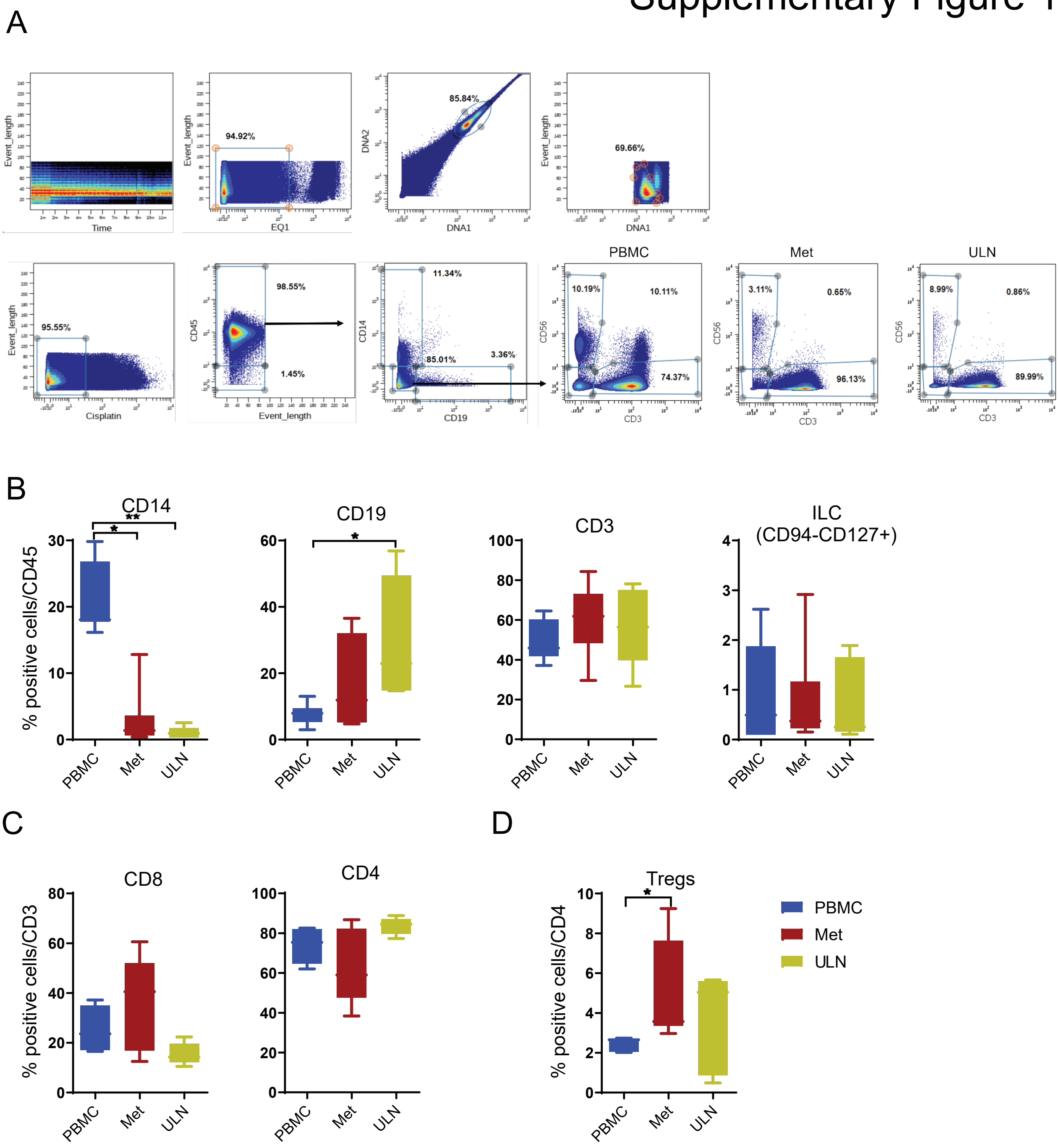

Supplementary Fig. S1. Mass cytometry analysis and main immune cell subsets in PBMC, Met and ULN from AM patient samples. (A) Gating strategy used to evaluate NK cells from PBMC, Met and ULN by Mass cytometry. (B) Percent of CD14+, CD19+, CD3+ T cells and ILC within CD45+ cells from PBMC, MLN and ULN is shown. (C) Percent of CD4+ and CD8+ T cells within the CD3+ gate. (D) Percent of regulatory T cells CD4+CD25^{+}CD127^{-} within CD4+ T cells. ILC3 cells were identified as CD45+CD14-CD19-CD3-CD127+CD16-. Bars represent Min and Max. Two-way ANOVA Test, Mixed effects Model with Turkey post-hoc test. *p<0.05, **p<0.01. n=11 PBMC, n=11 Met, n=7 ULN.
