# Supplementary material for: Memory-like Differentiation Enhances NK Cell Responses to Melanoma
Source: Clin Cancer Res. 2021 Jun 29;27(17):4859–69. doi: 10.1158/1078-0432.CCR-21-0851 (PMC8416927; doi:10.1158/1078-0432.CCR-21-0851)
Supplement: Supplementary Fig S2 — Infiltrating NK cells exhibit a tissue resident phenotype. [file 10780432ccr210851-sup-261875_2_supp_7159128_q91f91.pdf]

Supplementary Figure 2

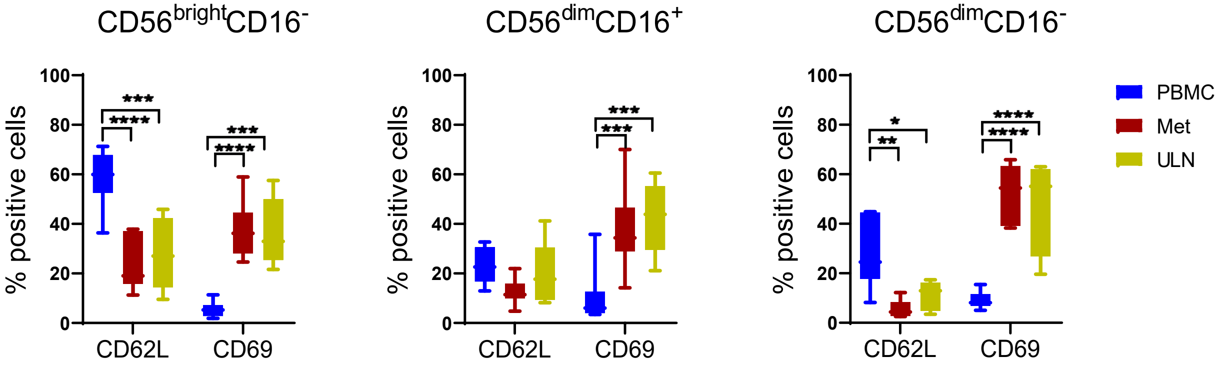

Supplementary Fig. S2. Infiltrating NK cells exhibit a tissue resident phenotype. Expression of the tissue resident markers CD62L and CD69 on (A) CD56^{bright}, (B) CD56^{dim}CD16^{+} and (C) CD56^{dim}CD16^{-} NK cells from PBMC, Met and ULN. Bars represent Min and Max. Two-way ANOVA Test, Mixed effects Model with Turkey post-hoc test. *$p<0.05$, **$p<0.01$, ***$p<0.001$, ****$p<0.0001$. n=11 PBMC, n=11 Met, n=7 ULN.
