# Supplementary material for: Memory-like Differentiation Enhances NK Cell Responses to Melanoma
Source: Clin Cancer Res. 2021 Jun 29;27(17):4859–69. doi: 10.1158/1078-0432.CCR-21-0851 (PMC8416927; doi:10.1158/1078-0432.CCR-21-0851)
Supplement: Supplementary Fig S3 — Phenotypic analysis of blood NK cells from AM patients and healthy donors. [file 10780432ccr210851-sup-261875_2_supp_7159129_qk1f91.pdf]

Supplementary Figure 3

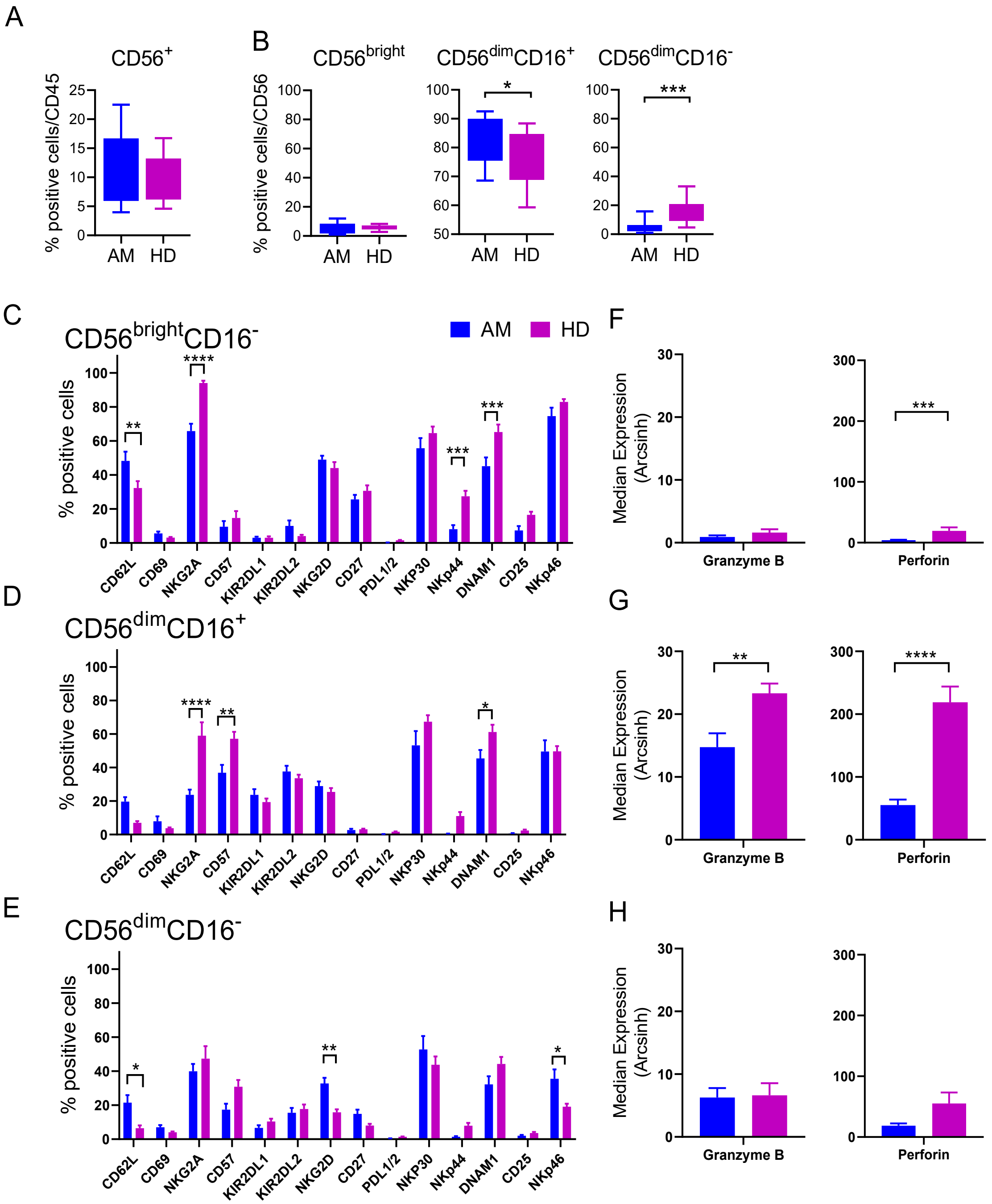

Supplementary Fig. S3. Phenotypic analysis of blood NK cells from AM patients and healthy donors. Frequency of circulating (A) NK cells and (B) NK cells subsets from advanced melanoma (AM) patients and healthy donors (HD). Expression of costimulatory, inhibitory and maturation markers on (C) $CD56^{bright}CD16^{-}$, (D) $CD56^{bright}CD16^{+}$ and (E) $CD56^{bright}CD16^{-}$ NK cells from AM and HD. Median expression of granzyme and perforin by (F) $CD56^{bright}CD16^{-}$, (G) $CD56^{dim}CD16^{+}$ and (H) $CD56^{dim}CD16^{-}$ NK cells. Bars represent Mean ± SEM. Two-way ANOVA with Sidak post-hoc test was used in C, D and E. Mann Whitney test was used to compare granzyme B and perforin in AM and HD in F, G, H. *p<0.05, **p<0.01, ***p<0.001, ****p<0.0001. n=11 AM and n=15 HD.
