# Supplementary material for: Memory-like Differentiation Enhances NK Cell Responses to Melanoma
Source: Clin Cancer Res. 2021 Jun 29;27(17):4859–69. doi: 10.1158/1078-0432.CCR-21-0851 (PMC8416927; doi:10.1158/1078-0432.CCR-21-0851)
Supplement: Supplementary Fig S4 — Cytokine production and degranulation of purified NK cells from normal donors [file 10780432ccr210851-sup-261875_2_supp_7159130_q81f92.pdf]

Supplementary Figure 4

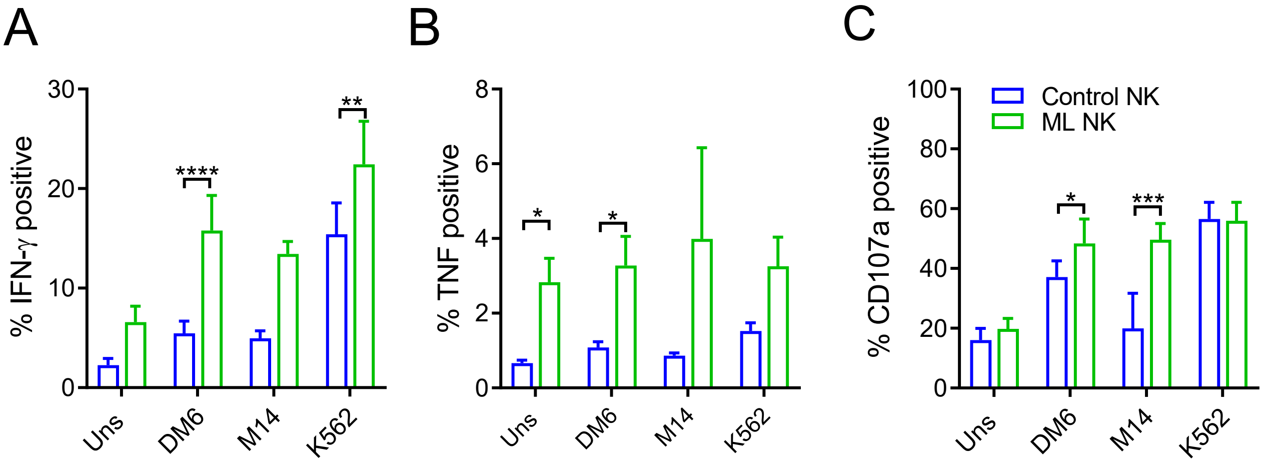

Supplementary Fig. S4. Cytokine production and degranulation of purified NK cells from normal donors. Purified NK from normal donors were activated with IL-12/IL-15/IL-18 (or IL-15 alone as control) for 16-18 hours as descried in Figure 2A. Control and ML NK cells were stimulated with DM6, M14 and K562 in a 6-hours functional assay and the frequency of IFN-γ, TNF, and degranulating (CD107a) cells was evaluated by flow cytometry. Bars represent Mean ± SEM. Two-way ANOVA with Sidak post-hoc test $*p<0.05$, $**p<0.01$, $***p<0.001$. n=2-8.
