# Supplementary material for: Memory-like Differentiation Enhances NK Cell Responses to Melanoma
Source: Clin Cancer Res. 2021 Jun 29;27(17):4859–69. doi: 10.1158/1078-0432.CCR-21-0851 (PMC8416927; doi:10.1158/1078-0432.CCR-21-0851)
Supplement: Supplementary Fig S5 — Immunohistochemistry staining of S100, Melan A and SOX10 in patient derived cell lines [file 10780432ccr210851-sup-261875_2_supp_7159131_q91f92.pdf]

Supplementary Figure 5

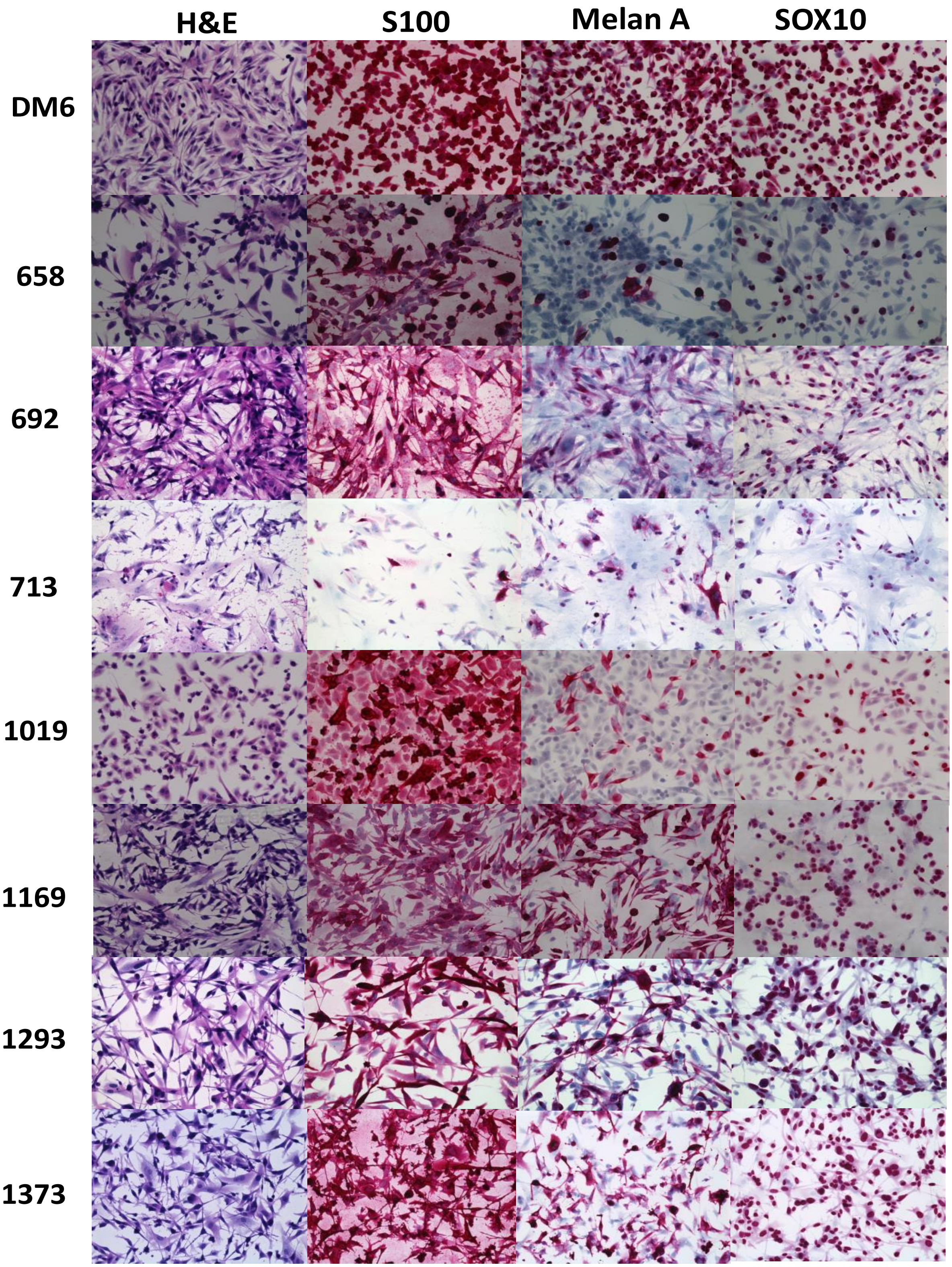

Supplementary Fig. S5. Immunohistochemistry staining of S100, Melan A and SOX10 in patient derived cell lines. Patient derived cell lines from seven AM patients were generated and used as autologous targets for functional assays using control and ML NK cells from the same patient. Cells were generated and stained as described in Supplemental material.
