# Supplementary material for: Memory-like Differentiation Enhances NK Cell Responses to Melanoma
Source: Clin Cancer Res. 2021 Jun 29;27(17):4859–69. doi: 10.1158/1078-0432.CCR-21-0851 (PMC8416927; doi:10.1158/1078-0432.CCR-21-0851)
Supplement: Supplementary Fig S6 — Cytokine production and degranulation of control and ML NK cells from AM patients and normal donors and effect of antibody blockade. [file 10780432ccr210851-sup-261875_2_supp_7159132_qd1f92.pdf]

Supplementary Figure 6

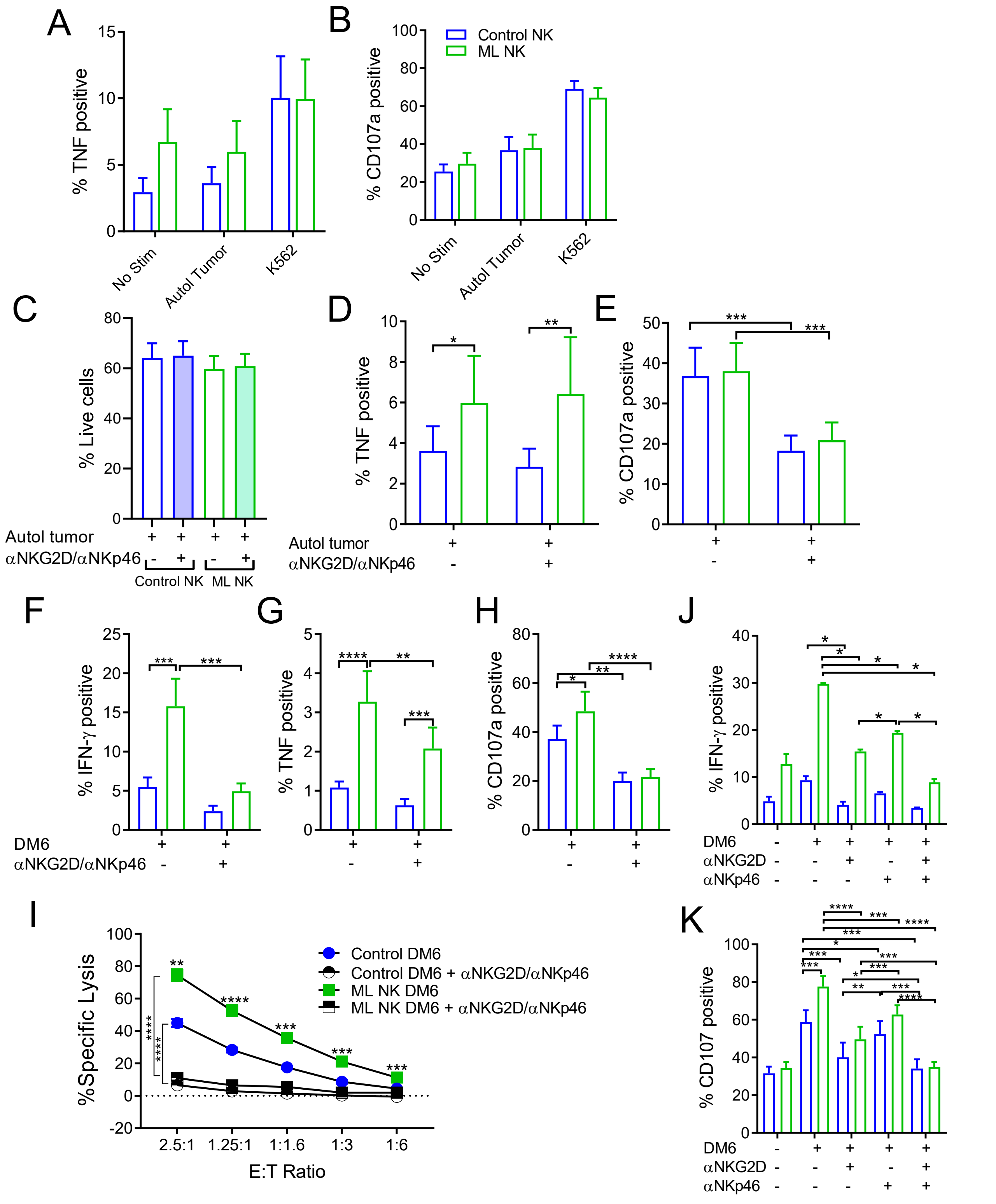

Supplementary Fig. S6. Cytokine production and degranulation of control and ML NK cells from AM patients and normal donors and effect of antibody blockade. (A) TNF production and (B) degranulation (CD107a+) of control and ML NK cells from AM in response to autologous melanoma targets. (C) Antibody blockade with αNKG2D and αNKp46 do not affect cell viability of control (blue) and ML NK (green) cells from AM patients. (D) TNF production and (E) degranulation of control (blue) and ML NK (green) cells from AM patients stimulated with autologous tumor in presence of αNKG2D + αNKp46 blocking antibodies. (F) IFN-γ, (G) TNF, (H) degranulation and (I) cytotoxicity of control (blue) and ML NK (green) cells from HD stimulated with DM6 in presence of αNKG2D/αNKp46 blockade. (J) IFN-γ and (K) degranulation (CD107a) of NK cells from HD in presence of single or combined αNKG2D and αNKp46 blockade. Figures A-E show PBMC-derived NK cells from AM patients (n=7) and F-K correspond to purified NK cells from HD (n=2-8). Bars represent mean ± SEM. Two-way ANOVA with Turkey posthoc analysis. *p<0.05, **p<0.01, ***p<0.001, ****p<0.0001.
